# Using an Active Sensor to Develop New Critical Nitrogen Dilution Curve for Winter Wheat

**DOI:** 10.3390/s20061577

**Published:** 2020-03-12

**Authors:** Jie Jiang, Cuicun Wang, Yu Wang, Qiang Cao, Yongchao Tian, Yan Zhu, Weixing Cao, Xiaojun Liu

**Affiliations:** National Engineering and Technology Center for Information Agriculture, MOE Engineering and Research Center for Smart Agriculture, Key Laboratory for Crop System Analysis and Decision Making, Ministry of Agriculture, Jiangsu Key Laboratory for Information Agriculture, Jiangsu Collaborative Innovation Center for Modern Crop Production, Nanjing Agricultural University, 1 Weigang Road, Nanjing 210095, China; 2019201009@njau.edu.cn (J.J.); wangcuicun@163.com (C.W.); wangyu_961016@163.com (Y.W.); qiangcao@njau.edu.cn (Q.C.); yctian@njau.edu.cn (Y.T.); yanzhu@njau.edu.cn (Y.Z.); caow@njau.edu.cn (W.C.)

**Keywords:** winter wheat, nitrogen diagnosis, grain yield, critical nitrogen dilution curve, active sensor

## Abstract

Critical nitrogen (N) dilution curves (CNDCs) have been developed to describe the dilution dynamic of N and to diagnose N status in plants. In this study, to develop a convenient alternative CNDC determination method, four field experiments involving different N rates (0–360 kg N ha^-1^) and six wheat varieties were performed at different eco-sites from 2014 to 2019. The normalised difference red-edge (NDRE) index extracted from the RapidSCAN CS-45 (Holland Scientific Inc., Lincoln, NE, USA) sensor was used as a driving factor instead of plant dry matter (PDM) to establish a new alternative winter wheat CNDC. The newly developed CNDC was described by the equation Nc = 0.90NDRE^−0.88^, when NDRE values were ≤ 0.19 and constant Nc = 3.81%, which was independent of the NDRE values. Compared to PDM-derived CNDC (R^2^ = 0.73) developed with the same dataset, a comparable precision was obtained using NDRE-derived CNDC (R^2^ = 0.76) and both CNDCs could accurately discriminate wheat N status. Moreover, the NDRE could be inexpensively and rapidly measured using the active sensor. The relationship between NDRE-derived CNDC and grain yield was also analysed to facilitate in-season N management, and the R^2^ value reached 0.79 and 0.87 at jointing and booting stages, respectively. The NDRE-based CNDC can be used to effectively diagnose wheat N status and as an alternative approach for non-destructive determination of crop N levels.

## 1. Introduction

The over-application of nitrogen (N) fertilizers is a common problem in modern crop production. Excess N causes a low efficiency of N use, increases a susceptibility to crop disease, promotes environmental pollution, and reduces grain quality for consumption and cooking [1,2]. Appropriate measures are therefore required to increase N use efficiency and reduce agricultural pollution, including the timely and accurate diagnosis of crop N status and site-specific N management. 

Plant-based analytical techniques such as chlorophyll meters [3] and remote sensing tools [4,5] are useful in assessing N deficiencies in crops, and can be used to optimize in-season N management to achieve high profitability and sustainability in crop production systems. However, these technologies are easily affected by cultivar and environmental differences, limiting their application for the detection of excessive crop N uptake [6]. As an exemplar, data collection from the chlorophyll meters or remote sensing tools are susceptible to saturation when N treatments are adequate [7,8]. Alternatively, critical N concentrations (Nc) were proposed to assess crop N status, defined as the minimum N concentration required for maximum crop growth [9]. Similar to the relationship between plant N concentrations (PNC) and plant dry matter (PDM), plant Nc decreases as the crop PDM increases in a negative power function termed the critical N dilution curve (CNDC) [10]. Plant CNDCs can be described by

(1) $$Nc=aDM^{-b}$$

where a represents the *Nc* for dry matter (*DM*) = 1 Mg ha^−1^ and b represents the decline in PNC with crop growth.

Greenwood et al. [11] defined two general CNDCs when the PDM exceeded 1 Mg ha^−1^ for C3 (*Nc* = 5.7*DM*^−0.5^) and C4 (*Nc* =4.1*DM*^−0.5^) species. Both the CNDCs show utility for plant N nutritional diagnoses, but their accuracy is limited by differences in histology, morphology and eco-physiological characteristics amongst species [12]. Hence, species-specific CNDCs have been established for winter rape [13], potato [14], cotton [15], maize [16], rice [17] and winter wheat [10]. Additionally, the CNDCs based on plant-specific growth indicators, such as leaf dry matter and the leaf area index (LAI), were also constructed to successfully discriminate crop N status [18,19]. Although plant biomass-based or specific growth indicator-based CNDCs show sufficient precision to assess the crop N status, certain limitations restrict its use in modern agricultural practices. For example, a large body of data is required to establish a CNDC, which is acquired through destructive sampling and time-consuming measurements. However, these procedures are beyond the ability of most farmers and the time required to obtain the information is unpractical [20]. To construct the CNDC more efficiently, other plant-related indices have been employed to replace the plant biomass or specific growth indicators in the allometric function. Wang et al. [21] developed a CNDC for japonica rice based on canopy coverage extracted from canopy images, which were easily obtained and provided extensive plant information. However, those analyses were image-based and required complex and time-consuming image processing [22,23]. This is challenging for farmers that lack specialized skills or equipment.

The vegetation index is defined as the combination of several spectral bands and simply and effectively represents plant growth status. The performance of vegetation index could be affected by some factors, such as soil background, water status, crop diseases and other nutrients [24,25]. Some studies take relevant methods, for example, the ‘useless pixels’ were filtered to remove the soil or water background pixels from original images [26], or the N-sufficient area was set to eliminate the influence of other than N nutrition [27], which partly improved the performance of vegetation indices. Previous studies have used a range of sensors to acquire the vegetation index for several staple crops (e.g. maize, soybean, rice and wheat), and found that the vegetation index is highly correlated with plant biomass, LAI, N status and grain yield [28,29,30,31]. The vegetation index only extracts the reflectance of each spectral band, which it calculates according to a specific formula. The vegetation index increases with increasing N fertilizer application rates, until a non-limiting N level is attained. The relationship between the vegetation index and PNC can also described by an allometric function. Therefore, the use of the vegetation index obtained from active sensors for the development of CNDC represents a rapid, convenient and accurate method for the assessment of crop N status.

Timely and accurate crop yield forecasts prior to harvest could guide producers for N fertilizer application and benefit grain-processing industries for the quantification of produce supply and market price under different ecological conditions and agronomic practices. Previous studies used a combination of normalized difference vegetation index (NDVI) and accumulated growing degree days to predict crop yield [32], but NDVI is likely to be saturated and the meteorological data are not readily available. Alternatively, crop growth simulation models could successfully estimate crop yields [33], using the inputs related to a wide range of environment variables, but they may not be easily accessible and be difficult for extension. Additionally, several efforts have been devoted to using the CNDC for in-season yield estimation and on-farm N dressing recommendation. The relationships between relative yield (RY) and average N nutrition index (NNI) during vegetative growth of maize and spring wheat have been used to predict grain yield [16,34]. However, crop growth stage is imperative for determining plant parameters to estimate grain yield and determination of the relationships at different growth stages will be beneficial for modelling crop N dynamics [35]. Further investigations are needed to evaluate whether the NNI at the critical N nutritional stages could predict winter wheat grain yield.

The objectives of this study were to: (1) develop a new CNDC using the vegetation index extracted from the RapidSCAN CS-45 active canopy sensor in winter wheat; (2) to assess the reliability of the new CNDC in determining winter wheat N status; (3) and to estimate grain yield using the new CNDC at the critical N nutritional stages. These results will provide a theoretical basis and new technical approach for the non-destructive determination of crop N status.

## 2. Materials and Methods

### 2.1. Experimental Design

**Figure 1:**
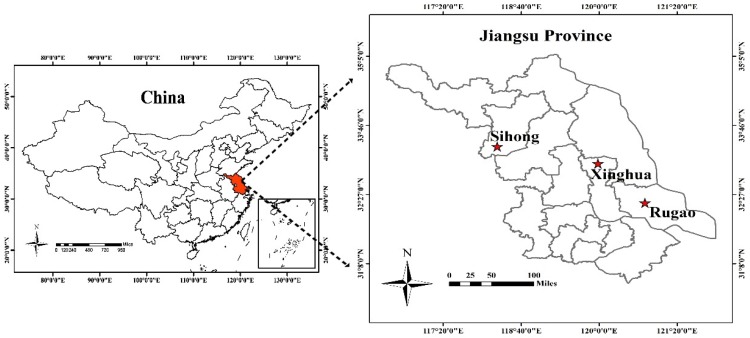
Study sites: wheat experiments were performed at Rugao, Sihong and Xinghua Experimental Stations in the Jiangsu province of China.

Experiment 1 (2014–2015) was conducted at Rugao Experimental Station (Figure 1; 32.27° N, 120.75° E) using three winter wheat varieties, Ningmai13, Huaimai20 and Yangfumai4, grown at four N levels: 0, 120, 225, and 330 kg N ha^−1^. Experiment 2 was conducted at Sihong Experimental Station (Figure 1; 33.37° N, 118.26° E) from 2015 to 2016, using two winter wheat varieties, Xumai30 and Huaimai20, at five N levels: 0, 90, 180, 270, and 360 kg N ha^−1^. Experiments 3 (2017–2018) and 4 (2018–2019) were conducted at Xinghua Experimental Station (Figure 1; 33.08° N, 119.98° E) using three winter wheat varieties, Zhenmai12, Yangmai23 and Ningmai13, grown at five N levels: 0, 90, 180, 270, and 360 kg N ha^−1^. Detailed information can be found in Table 1. 

A randomized complete block design with three replications was used in all experiments. Seeds were sown artificially and the row distance was 25 cm. Plants were grown at a density of 2.25 million seedlings ha^−1^. The granular urea was used as an N fertilizer in all experiments and applied in two batches: 50% before sowing and 50% at the wheat jointing stage. Based on soil analysis and the recommendations from the local agricultural department, 105 kg P_2_O_5_ ha^−1^ was applied prior to sowing in the form of Ca (H_2_PO_4_)_2_ and 135 kg K_2_O ha^−1^ was applied as two splits: 50% prior to sowing and 50% at the stem elongation stage. 

### 2.2. Spectral Data Collection

**Figure 2:**
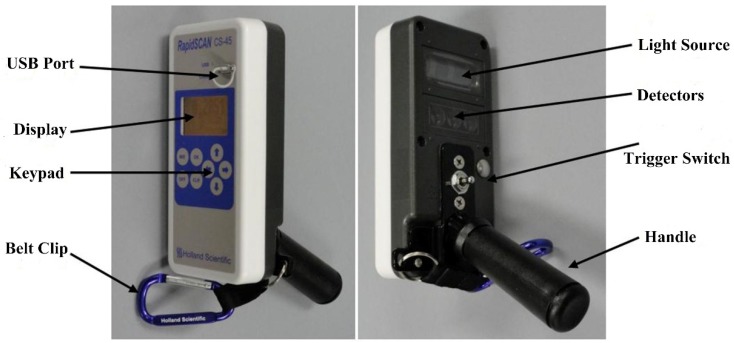
The RapidSCAN CS-45 canopy sensor.

The RapidSCAN CS-45 sensor (Figure 2) weighs 0.8 kg and has three fixed wavebands: red (R; 670 nm); red edge (RE; 730 nm); and near-infrared (NIR; 780 nm). The two default vegetation indices produced by the sensor include the normalized difference red edge (NDRE) and NDVI. The RapidSCAN CS-45 memory module automatically records the spectral reflectance (%) of the wheat canopy in each band, the default vegetation indices, and GPS data at a frequency of 2.5 Hz (one reading per 0.4 second). The sensor was held approximately 0.7–0.9 m above the winter wheat canopy and carried at a consistent speed to collect readings from three rows in each plot. The reflectance values were then averaged to represent the reflectance of each plot. The spectral reflectance values in the R, RE, and NIR bands were used to calculate 14 vegetation indices (detailed in Table 2).

### 2.3. Plant Sampling and Measurements

Upon the collection of spectral data, plants in the corresponding plots were destructively sampled. From each plot, 20 plants were randomly sampled and separated into stems and leaves. Sub-samples of the stems and leaves were oven-dried for 30 min at 105 °C to inhibit metabolic activity and then dried at 80 °C until a constant weight was achieved. The weight of each sub-sample was then recorded and the PDM was determined. The PNC was measured using the micro-Kjeldahl method [46]. Grain yield was determined in each plot by harvesting plants manually from three randomly identified areas of 1m^2^. The RY was calculated as the ratio of grain yield obtained for a given N rate with the highest grain yield among all N application rates.

### 2.4. Data Processing and Analysis

The methods used for the construction of the plant CNDC were previously proposed by Justes et al. [10]. Data collected from Experiments 2 and 3 were used for the calibration of the plant CNDCs. Data collected from Experiment 1 were used for the validation of the fitted CNDCs. Before the selection of the plant Nc, the NDRE and PDM values at each sampling date under different N treatments were compared using a one-way ANOVA (SPSS, Inc., Chicago, IL, USA, 2004) at a probability level of 5%. Following the determination of the plant Nc points of winter wheat, plant CNDCs were independently established based on the PDM (Equation (1)) or NDRE (Equation (2)).

(2) $$N_{C}=aNDRE^{-b}$$

Datapoints were selected from non-N-limiting treatments (N4 treatments in Experiments 2 and 3) for the determination of the maximum plant N curves (N_max_); the data points from N-limiting treatments (N0 treatments in Experiments 2 and 3) were used to construct the minimum plant N curves (N_min_).

The N nutritional index (NNI) of each sampling date was calculated according to the equation developed by Lemaire et al. [47]:

(3) $$NNI=\frac{N_{a}}{N_{c}}$$

where N_a_ is the actual measured N concentration and Nc is the critical N concentration determined by Equation (1) or (2). 

Data collected from Experiments 2 and 3 were used for the calibration of the relationships between RY and NNI. Data collected from Experiment 4 were used for the validation of the fitted functions. The coefficients for the determination (R²) of the relationship between the vegetation indices and PDM were calculated using Microsoft Excel (Microsoft Corporation, Redmond, WA, USA). The RY was expressed as a function of NNI, and the linear-plateau function was estimated using SPSS 18.0 (SPSS Inc., Chicago, Illinois, USA). Models with the highest R² were then selected. Additionally, model performance was evaluated using the root mean square error (RMSE) and relative error (RE), which were calculated as follows

(4) $$RMSE=\sqrt{\frac{1}{n}\times \overset{n}{\underset{1}{\sum }}{\left(P_{i}-Q_{i}\right)}^{2}}$$

(5) $$RE=100\times \sqrt{\frac{1}{n}\times \overset{n}{\underset{1}{\sum }}{\left(\frac{P_{i}-Q_{i}}{Q_{i}}\right)}^{2}}$$

where n is the number of samples, *Q_i_* is the observed value, and *P_i_* is the value derived from the model. All diagrams were plotted using GraphPad Prism 6 (GraphPad Software Inc., San Diego, CA, USA).

## 3. Results

### 3.1. Plant N Concentrations, Dry Matter and NDRE Under Different N Levels

**Figure 3:**
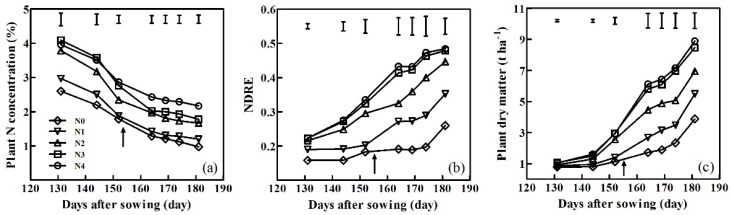
Changes in plant N concentrations (**a**), normalized difference red edge (NDRE) (**b**) and plant dry matter (PDM) (**c**) after sowing under different N treatments using ‘Xumai30’ in Experiment 2 as an example. Arrows indicate the days of topdressing N application. Vertical bars at each sampling date represent the LSD values (P ≤ 0.05).

The changes in PNC after sowing were similar under different N treatments for different cultivars and years. Here, we use ‘Xumai30’ in 2015-2016 as an example. PNC gradually declined from 131 to 181 days after sowing and increased with increasing N application, ranging from 0.97% to 4.09% (Figure 3a). The most rapid decrease in PNC for ‘Xumai30’ was observed from 131–164 days post-sowing. Following that period, the rate of PNC decreased under all N fertilizer treatments. Although the topdressing N fertilizer was applied at the pre-jointing stage, the supplied N did not influence the decreasing trend. 

**Figure 4:**
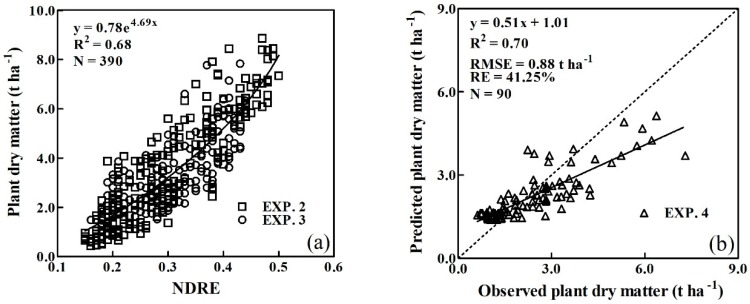
Calibration (**a**) and validation (**b**) results of the relationship between PDM and NDRE across all growth stages.

Based on the vegetation indices listed in Table 2, quantitative exponential relationships between them and the PDM were systematically analyzed. The top five vegetation indices described more than 67% of PDM variation across the entire growth stages and the validation data had R^2^ values ≥ 0.69, an RMSE of 0.88–0.91 t ha^-1^ and a RE of 41.25%–48.03% (Table 3). The NDRE was most closely associated with PDM (Figure 4), with R² values of 0.68 across all growth stages and validation R^2^ values of 0.70, an RMSE of 0.88 t ha^-1^ and a RE of 41.25%. The NDRE was therefore selected to derive the plant CNDC.

The NDRE and PDM had similar trends of days after sowing under different N levels in different cultivars and time periods. ‘Xumai30’ in the period 2015–2016 was used as an example. The NDRE and PDM increased gradually from 131 days after sowing, reaching a maximum at 181 days (Figure 3b,c). The N application rates significantly affected the NDRE and PDM (NDRE ranged from 0.16 to 0.48 and PDM ranged from 0.78 to 8.89 t ha^−^^1^) during the vegetative growth period. NDRE and PDM significantly increased from N0 to N2, but no significant differences were observed amongst N3 and N4 treatments.

### 3.2. Determination of the Critical N Dilution Curves

**Figure 5:**
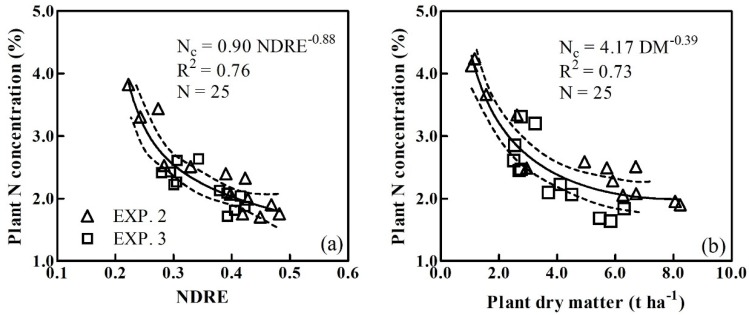
Critical N dilution curves derived from NDRE (**a**) and PDM (**b**). Dotted lines indicate 95% confidence intervals.

Based on the quantitative analysis of the relationship between vegetation indices and PDM, the NDRE was selected to derive the plant CNDC. The data collected on sampling dates from experiments 2 and 3 were used to determine the NDRE-derived plant CNDC following the computational procedures of Justes et al. [10]. Meanwhile, the same datasets and methods were used to construct the PDM-derived plant CNDCs. Amongst the 26 plant Nc points, a single point with an NDRE value less than 0.2 was excluded from the CNDC. Therefore, only 25 critical points were assessed, with NDRE values ranging from 0.22 to 0.48, that were used to establish the NDRE-derived CNDC (Figure 5a). Similarly, a single point with PDM values less than 1 t ha^−1^ were excluded from the regression analysis. The remaining 25 critical points with PDMs ranging from 1.26 to 8.23 t ha^−1^, were used to establish the PDM-derived CNDC (Figure 5b). Declining trends in the plant Nc values were observed with increasing NDRE or PDMs, which could be fitted by the following relationships for NDRE- (Equation (6) and Figure 5a) and PDM-derived (Equation (7) and Figure 5b) plant CNDCs:

(6) $$N_{c}=0.90NDRE^{-0.88}$$

(7) $$N_{c}=4.17DM^{-0.39}$$

The developed PDM-derived CNDC accounted for 73% of the total variance. The NDRE-derived CNDC achieved an R^2^ value of 0.76.

**Figure 6:**
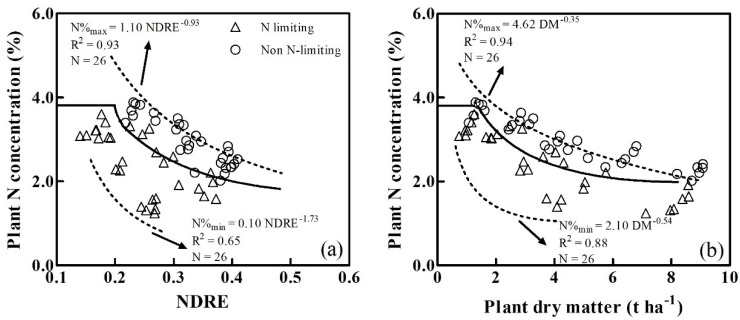
Validation of the critical N dilution curves established by NDRE (**a**) and PDM (**b**). Dashed lines represent the maximum and minimum boundaries of plant N concentrations.

The plant CNDC could not be applied to the NDRE or PDM values in the lower ranges observed during the earlier growth stages, and the NDRE values, which ranged from 0.16 to 0.22, but the PDM values ranging from 0.62 to 1.05 t ha^-1^, could, however, be used to establish the constant plant Nc. In this case, a constant PNC value of 3.81% was used for the NDRE- and PDM-derived CNDCs. From the dilution curves, the PNC and constant PNC intersected at an NDRE of 0.19 (Figure 6a) and at a PDM of 1.26 t ha^−1^ (Figure 6b). 

Data points from non-N-limiting treatments (N4 treatment) and from N-limiting treatments (N0 treatment) in Experiments 2 and 3 were used to develop two limit curves. 

The maximum N and minimum N dilution curves in Figure 6a were:

(8) $$N_{max}=1.10NDRE^{-0.93}$$

(9) $$N_{min}=0.10NDRE^{-1.73}$$

The maximum N and minimum N dilution curves in Figure 6b were:

(10) $$N_{max}=4.62DM^{-0.35}$$

(11) $$N_{min}=2.10DM^{-0.54}$$

### 3.3. Validation of the Critical N Dilution Curves

The NDRE-derived and PDM-derived CNDCs were tested against independent datapoints (N = 60) from Experiment 1, conducted during the 2014–2015 season. The datapoints from N0 and N1 treatments were characterized as N-limiting, whilst the datapoints from N2 and N3 treatments were characterized as non-N-limiting. In general, the NDRE- and PDM-derived CNDCs effectively discriminated the N-limiting and non-N-limiting groups (Figure 6), although several points could not be discriminated. As shown in Figure 6, all datapoints from the N-limiting treatments were close to or below the plant CNDCs, whilst those from non-N-limiting treatments were close to or above the plant CNDCs.

### 3.4. Variability of NNI During Winter Wheat Development

**Figure 7:**
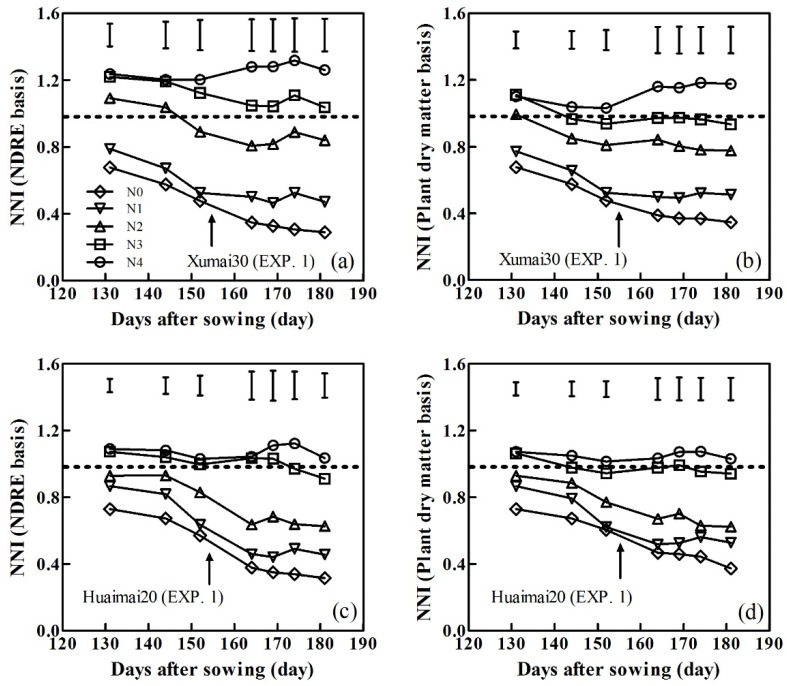
N nutrition index (NNI) calculated from the NDRE at different sampling dates for ‘Xumai30 (EXP. 1)’ (**a**) and ‘Huaimai20 (EXP. 1)’ (**c**). The NNI was calculated based on the PDM at different sampling dates for ‘Xumai30 (EXP. 1)’ (**b**) and ‘Huaimai20 (EXP. 1)’ (**d**). Arrows indicate the days of topdressing N application. Vertical bars at each sampling date represent the LSD values (P ≤ 0.05).

NNI values were independently calculated based on NDRE- and PDM-derived CNDCs. Here, we use ‘Xumai30’ and ‘Huaimai20’ in the period 2015–2016 as examples (Figure 7). Similar trends were observed between the NNIs and the two cultivars. Following spring regrowth, both NNIs showed declining trends until topdressing N applications were performed in the two cultivars, that led to modest increases in the NNI values. Thereafter, the NNI decreased as the winter wheat development and leaves senesced. Both types of NNI increased with increasing N rates; NNI was calculated based on the NDRE-derived CNDC ranging from 0.29 to 1.32 in cultivar ‘Xumai30’ (Figure 7a) and from 0.32 to 1.12 in cultivar ‘Huaimai20’ (Figure 7c) during the growing season. NNIs were calculated based on the PDM-derived CNDC that ranged from 0.35 to 1.18 in cultivar ‘Xumai30’ (Figure 7b) and from 0.37 to 1.08 in cultivar ‘Huaimai20’ (Figure 7d) during the growing season. Both types of NNI values for the N3 treatment in the two cultivars were close to 1, indicating that the levels of N were optimal.

### 3.5. The Relationship Between Grain Yield and CNDC

**Figure 8:**
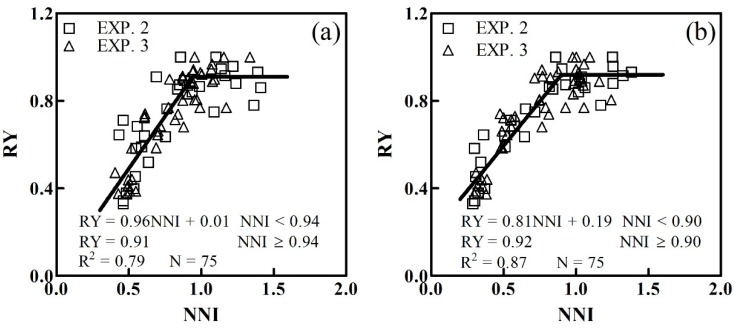
Prediction models for estimation of relative yield (RY) from NNI at the jointing (**a**) and booting (**b**) stages.

To facilitate the application of NDRE-derived CNDC for in-season yield estimation, the quantitative linear-plateau function relationships between RY and NNI were systematically analyzed at jointing and booting stages (Table 4). At the jointing stage, the NNI described 79% of the RY variation (Figure 8a). The validation data from Experiment 4 showed that the linear-plateau function had R^2^ values of 0.76, an RMSE of 0.09 and an RE of 12.50%. At the booting stage, the NNI showed an improved performance, which explained 87% of the NNI variation (Figure 8b). Validation results showed that the linear-plateau function had R^2^ values of 0.80, an RMSE of 0.07 and an RE of 9.56%. 

## 4. Discussion

### 4.1. Variability in Plant Nitrogen Concentrations and NDRE

The PNC declined over time as previously reported [11]. This phenomenon is interpreted as a direct result of plant ageing and typically related to phenology, leading to large differences in the PNC between species within a given environment. The different environments vary for a given species [48]. This study demonstrated a significant variation in PNC over time due to the N dilution effect and soil N supplement under different N application rates (0-360 kg N ha^-1^). ‘Xumai30’ in 2015-2016 was used as an example. The PNC ranged from 0.97% to 4.09% under different N treatments during vegetation growth (Figure 3a). The N dilution phenomenon was mainly attributed to (1) the self-shading of leaves, which induced non-uniform leaf N content from the upper canopy leaves with high N concentrations to the shaded leaves with low N concentrations [49] and (2) a decrease in PNC with increasing stem/leaf ratios during crop development [50]. 

This study also demonstrated a significant variation in NDRE over time. ‘Xumai30’ in the period 2015-2016 was used as an example. NDRE ranged from 0.16 to 0.48 under different N treatments during vegetation growth (Figure 3b). Cao et al. [28] similarly demonstrated that the NDRE derived from the Crop Circle ACS-470 showed a significant variation in rice prior to the heading stage and a favorable good correlation between the NDRE and PDM (R^2^=0.54) was observed. The NDRE (R^2^ = 0.68) demonstrated a better relationship with PDM compared to all vegetation indices extracted from the RapidSCAN CS-45 active sensor. Li et al. [51] also indicated that the NDRE (R^2^ = 0.77) showed an improved performance for rice leaf dry matter estimations, which were similar to those employed in this paper. Previous studies indicated that NDRE could effectively mitigate the saturation effects under high biomass conditions, showing a high performance for LAI and N status estimations [52]. This study showed that winter wheat PNC decreased as the NDRE increased, independently of a sufficient N supply. This trend was obvious from maximum (Nmax=1.10NDRE^-0.93^) and minimum (Nmin=0.10NDRE^-1.73^) N dilution curves (Figure 6a). These allometric functions were similar to the relationship between PDM and PNC [53].

### 4.2. Comparison of the CNDCs based on the NDRE and Plant Dry Matter

**Figure 9:**
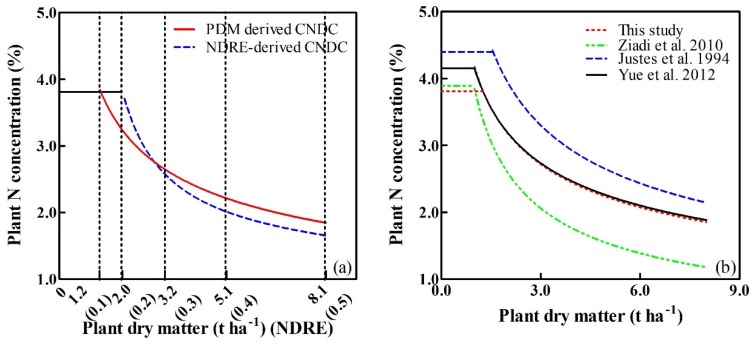
(**a**) Comparison of critical N dilution curves based on winter wheat NDRE and PDM. Intervals on the horizontal axis for NDRE were transformed so that the NDRE values coincided with the exponentially increasing PDM. (**b**) Comparison of different critical N dilution curves based on PDM in wheat.

The quantitative relationship between the PDM and NDRE values, PDM = 0.78e^4.69NDRE^ (Figure 4a), was used to convert the NDRE to PDM. Then, the intervals for NDRE on the horizontal axis could be adjusted to fit to the exponentially increasing PDM (Figure 9a). Thus, the NDRE- and PDM-derived CNDCs could be directly compared. The adjusted horizontal axis showed that NDRE increments (0.1–0.2) had the lowest levels of PDM accumulation (1.2–2.0 t ha^−1^). Along with the increases in NDRE, the distance for the same increments of NDRE increased, indicating that similar increments of NDRE required higher levels of PDM accumulation during the later growth stages. The self-shading of leaves was more pronounced with crop development, and the increment in the spectral index gradually reduced [54]. 

Generally, two types of CNDCs with similar trends were observed, in which small differences in the development trends of the two curves may have been due to errors in the PDM estimation model. During the early stages, PNC maintained a constant value, mainly due to the slowly increasing PDM and the lack of light competition amongst the isolated plants [13]. The same constant PNC values of 3.81% were used for NDRE- (NDRE less than 0.19) and PDM-derived (PDM less than 1.26 t ha^−1^) CNDCs. The NDRE-derived CNDC had a longer constant PNC, mainly due to the NDRE increasing more rapidly than the PDM during the early growth stages of winter wheat. This was in agreement with previous studies in which canopy-coverage-based CNDC had a longer constant PNC than that of PDM-derived CNDC [21]. The developed NDRE-derived CNDC accounted for 76% of the total variance, which was a comparable accuracy for that observed in the PDM-derived CNDC, with an R^2^ value of 0.73. The determination of the PDM-derived CNDC requires a large quantity of data acquired through a series of complex procedures, including destructive sampling, plant biomass measurements and the assessment of N content, which may have led to large errors. Moreover, professional measurement equipment is required to obtain the necessary data, which is beyond the ability and expertise of most farmers. In this study, the spectral index NDRE replaced biomass as the driving factor to construct the CNDC of winter wheat. The measurement process for canopy spectral data was relatively simple and stable, reducing sampling errors, improving the modeling accuracy, and greatly reducing the time required for destructive sampling, promoting the practical application of the CNDC. Wang et al. [21] also established a new rice CNDC (R^2^=0.88) based on the canopy coverage extracted from digital images and found a slightly higher modeling accuracy than PDM-derived CNDC (R^2^=0.86). Two types of CNDCs showed a similar performance in discriminating the rice N status, but measurements of the canopy coverage were more cost-effective and convenient than those of the PDM. However, the processing of these image-based data, which included image capturing, splicing, geometric correction, and the extraction of relevant spectral information, was complex and often time-consuming, particularly when the data were collected over large areas. Data processing also requires high-performance computers, specific software, and highly trained technical staff [55,56]. The reflectivity of the three bands (R, RE and NIR) could be directly exported from RapidSCAN CS-45 in the absence of pre-processing upon measurement completion. The vegetation index could be calculated from a simple formula, for example, NDRE = (NIR – RE) / (NIR + RE). Moreover, the RapidSCAN CS-45 active canopy sensor used in this study was not influenced by ambient illumination, as it contains an internal polychromatic light source. Therefore, NDRE acquisition using this sensor is unaffected by the sampling time or weather, and data on crop growth can be collected in real time. In general, the NDRE-derived CNDC was suitable for use by non-scientists for the diagnosis of crop N status during the actual production period.

### 4.3. Comparison of the CNDCs based on the PDM

The determination of CNDCs (*Nc* = a*DM*^−b^) has been realized for different wheat species in different regions (Figure 9b). The coefficient “a” represents PNC when PDM is 1 t ha^−1^, whilst coefficient “b” represents the factor of dilution describing the relationship between PNC and PDM. Our calculated plant CNDC model was *Nc* = 4.17*DM*^−0.39^, with constant plant Nc values of 3.81% prior to the PDM reaching 1.26 t ha^−1^. The CNDC derived from spring wheat in Canada displayed a similar “a” value (a = 3.85) but a higher coefficient “b” value (b = 0.57), resulting in a more rapid decline in PNC with crop growth [34]. The reasons for this discrepancy may be related to the differences in the type of wheat (winter vs. spring), ecological area (Canada vs. China) and growth patterns, in which winter wheat cultivars have an extended vegetative growth period due to vernalization requirements [57]. The CNDC developed in France *Nc* = 5.35*DM*^−0.44^ [10] is widely recognized as a reference curve for winter wheat and had a higher R^2^ (0.98) than that achieved in this study (R^2^ = 0.73). A total of 21 critical points that fulfill the defined statistic criteria out of 120 points were selected to establish the CNDC by Juetes et al. [10], while 25 out of 26 points were used to establish the CNDC in this study. The CNDC created in this study was lower than that of Justes et al. [10], with these discrepancies likely being due to the lower spring temperature and higher rainfall in Northern France vs. China, leading to a relatively longer growing season [58]. Winter wheat in Northern France therefore has a greater opportunity to accumulate N compared to Eastern China. The coefficients “a” and “b” in CNDC (*Nc* = 4.15 *DM*^−0.38^) established by Yue et al. [59] were close to those of CNDC in this study. These similarities were attributed to the comparable climatic conditions and fertilization practices in the same country. Seeding rate was different among different eco-sites with diverse latitudes; for example, approximate 2.50 million seedlings ha^−1^ were applied for the research in Canada, while plants grown at a density of 2.25 million seedlings ha^−1^ in this study, and these varieties have a strong tillering capacity. Seeding rate would have impacted tillering and self-shading [60,61], which also led to the difference among the above CNDCs.

The aforementioned discrepancy in several CNDCs of wheat is attributed to the differences in climatic conditions, cultivars, seed rate and production management. Previous studies compiled this information from various ecological areas to build a more adaptable CNDC. Five local major cultivars were used in this study that displayed similar growth developmental processes. It was therefore possible to develop reliable CNDCs using a combination of these data. Further studies are required to assess the applicability of CNDCs in larger eco-regions.

### 4.4. Application of CNDC for in-Season Yield Estimation

In-season estimation of winter wheat grain yield is fundamental for N fertilizer application. The relationships between RY and NNI on the basis of CNDC in this study can help in developing winter wheat yield prediction models for in-season estimation of grain yield. Tahir Ata-Ul-Karim et al. [35] established rice yield estimation models based on CNDC and found the highest modeling accuracy in the panicle initiation (R^2^ =0.96) and booting (R^2^ =0.96) stages. In this study, NNI-based yield estimation models were established at jointing (R^2^ = 0.79) and booting (R^2^ = 0.87) stages, which are the critical topdressing N stages for wheat production. The N accumulation during this period could effectively contribute to grain filling in wheat. Therefore, it is advisable to confirm sufficient N accumulation prior to the onset of reproductive stages. Furthermore, the algorithms based on CNDC can be coupled with some crop growth simulation models applicable to winter wheat, such as WheatGrow and AFRCWHEAT2 [62,63]. These will help to provide an accurate prediction of the growth and grain yield in winter wheat production. The nondestructive estimation of NNI can promote the practical implementation of this approach, for example, GreenSeeker vegetation indices were moderately related to maize NNI at V7–V8 (R^2^ = 0.51–0.55), V9-V10 (R^2^ = 0.44–0.45) and across the growth stages (R^2^ = 0.33–0.36) [29]. Cao et al. [64] also demonstrated that winter wheat NNI could be accurately estimated at Feekes growth stages 4–7 (R^2^ = 0.52–0.54), 8–10 (R^2^ = 0.55–0.64) and across stages (R^2^ = 0.44–0.47). The RapidSCAN CS-45 active canopy sensor used in this study also be a practical and rapid tool that can achieve this aim at any time of the day and under cloudy conditions. The RapidSCAN CS-45 runs on Pseudo Solar Reflectance (PSR) technology that permits height-independent measurements (0.3–3 m; www.hollandscientific.com). Thus, the RapidSCAN CS-45 active sensor can be carried on a low-altitude platform to collect data at a broad spatial scale [65]. Then, the yield estimation can be more convenient and efficient, and this should be considered in future studies.

## 5. Conclusions

In this study, a novel CNDC (*Nc* = 0.90NDRE^−0.88^, constant *Nc* = 3.81% at NDRE values ≤ 0.19) was developed based on the NDRE extracted from the RapidSCAN CS-45 active sensor. Compared with PDM-derived CNDC (R^2^ = 0.73) developed with the same datasets, a comparable precision was observed for the NDRE-derived CNDC (R^2^ = 0.76) and both CNDCs could accurately discriminate plant N status. In addition, NDRE measurements were more convenient and non-destructive than those of PDM. The relationship between NDRE-derived CNDC and grain yield was quantified to facilitate in-season N management, and the R^2^ value reached 0.79 and 0.87 at jointing and booting stages, respectively. The newly developed CNDC provides a quick approach and alternative tool to assess plant N nutrition status for guiding precision N management in winter wheat. 

## Figures and Tables

**Table 1:** Description of the field experimental conditions.

Experiment No.	Location	Plot Size (m^2^)	Cultivar	N Rate (kg ha^−1^)	Sampling Stage (date)	Soil Classification
Experiment 1 2014–2015	Rugao (32.27° N, 120.75° E)	30 (5 m × 6 m)	Ningmai13 Huaimai20 Yangfumai4	0 (N0) 120 (N1) 225 (N2) 330 (N3)	Spring re-growth (9 February) Pre-jointing (8 March) Mid-jointing (19 March) Post-jointing (28 March) Booting (8 April)	Soil type = Loam soil Soil pH = 6.40 OM = 23.55 g kg^−1^ Total N = 1.55 g kg^−1^ Available P = 44.80 mg g^−1^ Available K = 110.50 mg g^−1^
Experiment 2 2015–2016	Sihong (33.37° N, 118.26° E)	30 (5 m × 6 m)	Xumai30 Huaimai20	0 (N0) 90 (N1) 180 (N2) 270 (N3) 360 (N4)	Spring re-growth (3 March) Spring re-growth (16 March) Pre-jointing (23 March) Mid-jointing (5 April) Post-jointing (10 April) Booting (15 April) Heading (22 April) Harvest (3 June)	Soil type = Clay soil Soil pH = 6.56 OM = 26.3 g kg^−1^ Total N = 2.91 g kg^−1^ Available P = 43.12 mg g^−1^ Available K = 89.23 mg g^−1^
Experiment 3 2017–2018	Xinghua (33.08° N, 119.98° E)	63 (7 m × 9 m)	Zhenmai12 Yangmai23 Ningmai13	0 (N0) 90 (N1) 180 (N2) 270 (N3) 360 (N4)	Pre-jointing (22 March) Mid-jointing (1 April) Post-jointing (8 April) Booting (15 April) Harvest (2 June)	Soil type = Loam soil Soil pH = 6.61 OM = 21.26 g kg^−1^ Total N = 1.71 g kg^−1^ Available P = 41.06 mg g^−1^ Available K = 108.61 mg g^−1^
Experiment 4 2018–2019	Xinghua (33.08° N, 119.98° E)	63 (7 m × 9 m)	Zhenmai12 Yangmai23 Ningmai13	0 (N0) 90 (N1) 180 (N2) 270 (N3) 360 (N4)	Jointing (22 March) Booting (4 April) Harvest (29 May)	Soil type = Loam soil Soil pH = 6.61 OM = 21.26 g kg^−1^ Total N = 1.71 g kg^−1^ Available P = 41.06 mg g^−1^ Available K = 108.61 mg g^−1^

**Table 2:** Vegetation indices used for the RapidSCAN CS-45 sensor.

Index	Formula	Reference
Normalised difference vegetation index (NDVI)	(NIR – R)/(NIR + R)	[36]
Normalised difference red-edge (NDRE)	(NIR – RE)/(NIR + RE)	[37]
Red edge soil-adjusted vegetation index (RESAVI)	1.5 × [(NIR – RE)/(NIR + RE + 0.5)]	[38]
Difference vegetation index (DVI)	NIR – R	[39]
Soil-adjusted vegetation index (SAVI)	(1 + L)(NIR – R)/(NIR + R + L); L = 0.5	[24]
Red edge ratio vegetation index (RERVI)	NIR/RE	[40]
Perpendicular vegetation index (PVI)	(NIR + 1.05R – 0.03)/SQRT(1 + 1.05^2^)	[39]
Red edge difference vegetation index (REDVI)	NIR – RE	[36]
Ratio vegetation index (RVI)	R/NIR	[41]
Red edge wide dynamic range vegetation index (REWDRVI)	(a × NIR – RE)/(a × NIR + RE); a = 0.12	[42]
Optimised vegetation index 1 (VIopt1)	100 × (lnNIR – lnRE)	[40]
Transformed vegetation index (TVI)	SQRT((NIR – R)/(NIR + R) + 0.5)	[43]
Optimised soil-adjusted vegetation index (OSAVI)	(NIR – R)/(NIR + R + 0.16)	[44]
Reflection in red-edge (RRE)	(NIR + R)/2	[45]

**Table 3:** Calibration and validation results for the relationship between plant dry matter (PDM) and the top 5 vegetation indices across all growth stages.

Vegetation Indices	Calibration		Validation		
	Regression Equation	R²	R²	RMSE (t ha^-1^)	RE (%)
NDRE	*y* = 0.78e^4.69*x*^	0.68	0.70	0.88	41.25
RESAVI	*y* = 0.78e^3.14*x*^	0.68	0.70	0.88	41.30
VIOPT1	*y* = 0.86e^0.02*x*^	0.67	0.69	0.88	43.28
REWDRVI	*y* = 217.18e^6.70*x*^	0.67	0.69	0.91	48.07
REDVI	*y* = 1.07e^0.06*x*^	0.67	0.69	0.91	48.03

**Table 4:** Calibration and validation results for the relationship between relative yield (RY) and NNI at the jointing and booting stages.

Growth Stage	Calibration			Validation		
	Regression Equation		R²	R²	RMSE	RE (%)
Jointing stage	*y* = 0.96*x* + 0.01	*x* < 0.94	0.79	0.76	0.09	12.50
	y = 0.91	*x* ≥ 0.94				
Booting stage	*y* = 0.81*x* + 0.19	*x* < 0.90	0.87	0.80	0.07	9.56
	y = 0.92	*x* ≥ 0.90				
